# Translate to divide: сontrol of the cell cycle by protein synthesis

**DOI:** 10.15698/mic2015.04.198

**Published:** 2015-03-20

**Authors:** Michael Polymenis, Rodolfo Aramayo

**Affiliations:** 1 Department of Biochemistry and Biophysics, Texas A&M University, College Station, TX 77843, USA.; 2 Department of Biology, Texas A&M University, College Station, TX 77843, USA.

**Keywords:** protein synthesis, ribosome biogenesis, ribosome profiling, START, translational control

## Abstract

Protein synthesis underpins much of cell growth and, consequently, cell multiplication. Understanding how proliferating cells commit and progress into the cell cycle requires knowing not only which proteins need to be synthesized, but also what determines their rate of synthesis during cell division.

## INTRODUCTION

Experiments with proliferating populations of microbial strains, animal or plant cell lines, have rigorous expectations. Under the same culture conditions, cells ought to have the same properties and composition in every single experiment. The basic “metrics” of proliferating cells remain constant, even after many rounds of cell division 1,2. These metrics include cellular mass and volume, and macromolecular composition 1,3. The constancy of such parameters reflects the fundamental ability of cells to coordinate their growth with their division 1,4,5. Balancing cell growth with cell division determines the overall rates of cell proliferation 4,5,6,7,8. Despite the obvious significance of this phenomenon, how cells manage to coordinate their growth with their division remains largely mysterious.

Proteins are often the most abundant macromolecules in proliferating cells. For example, in steady-state cultures of the budding yeast *Saccharomyces cerevisiae*, the protein content ranges from 35% to 44% of all macromolecules, depending on culture conditions 3. Furthermore, much of the proteome (>20%) is dedicated to making ribosomes and translation factors, enabling cells to make more proteins 9. On top of that, making ribosomal components and assembling them into functional ribosomes involves a dizzying array of molecular players and cellular processes 10,11,12. Consequently, protein synthesis is viewed as a fundamental measure of cell growth. Decades ago, a founding father of cell cycle studies put it this way: “No sensible interpretation of cell growth can be made without a knowledge of the overall pattern of protein synthesis” 13.

In the following sections, we discuss the interplay of protein synthesis and cell division. Examples of translational control in embryonic and meiotic cell divisions have been covered comprehensively elsewhere 14,15. Here, the focus is on mitotic cell division and specifically on the G1 phase of the cell cycle, when cells commit to a new round of cell division. The examples discussed are mainly, but not exclusively, from the budding yeast *S. cerevisiae*. The discussion centers on un-perturbed, continuously dividing cells, and the impact of genetic, nutritional or chemical perturbations.

**Figure 1:**
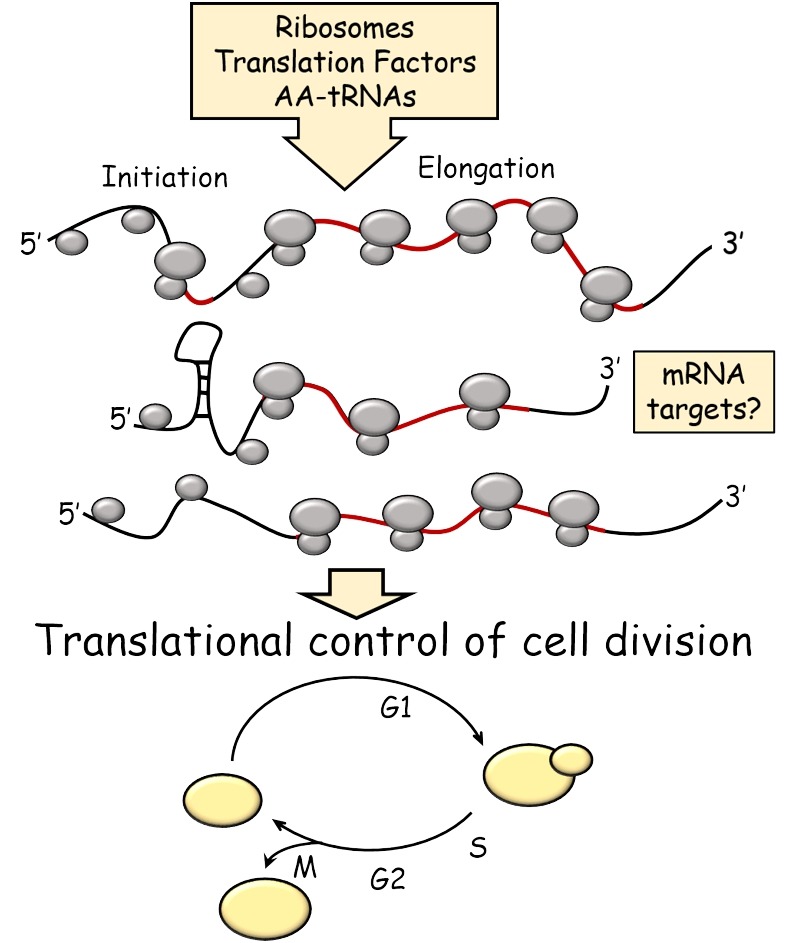
FIGURE 1: Schematic overview of the topics covered in this review. Open reading frames (ORFs) are shown in red.

## OVERALL PATTERN OF PROTEIN SYNTHESIS IN THE CELL CYCLE

In animal cells, protein synthesis is much lower in mitosis than in other cell cycle phases 16,17,18. Mechanisms that enable translation of specific mRNAs in animal cells undergoing mitosis have been reviewed elsewhere 15,19. In contrast to the mitotic block in protein synthesis in animal cells, early studies indicated that budding yeast cells synthesize proteins, including ribosomal proteins, continuously during the cell cycle 20,21,22,23. These experiments relied mostly on incorporation of labeled amino acids into polypeptides, which were then visualized after electrophoresis 21,22,23. Hence, those early experiments sampled abundant, constitutively expressed proteins that make up the vast majority of the proteome 9,24,25,26. Obviously, transcriptional waves drive periodic synthesis of hundreds of proteins in the cell cycle 27,28. Nonetheless, the bulk of cellular protein synthesis appears to proceed at an exponentially increasing rate in the cell cycle 21,22. This conclusion was reinforced by monitoring the accumulation of constitutively expressed fluorescent proteins in single cells 29. In addition, continuous monitoring of cell volume supports an exponential mode of increase in the cell cycle 30. Therefore, it appears that budding yeast cells make proteins and grow exponentially. Based on buoyant mass as a metric of cell growth, the same can be said about the growth of diverse types of cells, from bacteria to mouse lymphoblasts, with heavier cells growing faster than lighter cells 31. However, whether or not the growth of animal cells is exponential is still controversial 32,33,34.

An exponential mode of protein synthesis and growth is consistent with the existence of active mechanisms that sense some growth metric, perhaps somehow related to protein synthesis 7. Such mechanisms would enable cells to monitor their growth and commit to a new round of cell division once their growth requirements are met 6,7. As a result, cells that are born small stay longer in the G1 phase, until they grow enough to commit into and initiate a new round of cell division 4,5,6,7,35. In yeast, the point of commitment to a new round of cell division is called START 5. START is marked molecularly by nuclear eviction of the Whi5p repressor 29,36,37, a protein that functions analogously to the retinoblastoma gene product of animal cells 38,39. Once cells pass through START in late G1, they will initiate and complete their division even if they encounter growth limitations 4,5,35. To summarize simply, it seems that the bigger yeast cells get, the faster they make proteins and grow, propelling them to divide. This simple concept raises a series of key questions: What determines the rate of protein synthesis? How can the rate of protein synthesis be altered and what would the effects of such alterations be on the cell cycle? What are the RNA targets of translational control that affect cell cycle progression?

## INITIATE TO START? 

The rate of synthesis of any given protein depends on not only the concentration but also the translational efficiency of its mRNA. Discrepancies between the two parameters underpin translational control. It is often stated that control of translation in eukaryotic cells is exercised mainly at the initiation step, when ribosomes are recruited to mRNA 19. As discussed in subsequent sections, additional layers of control may also change the rate at which proteins are made. Nonetheless, the initiation step remains a key control point of translation 40,41. Ribosomal recruitment in eukaryotes usually involves recognition of a cap structure at the 5’-end of the mRNA. The small (40S) ribosomal subunit loaded with initiator Met-tRNA and with the contribution of various initiation factors begins scanning the 5’-UTR of the mRNA for an AUG (or near-cognate start codons). In the process, it has to navigate past the secondary structure of the 5’-UTR 42 or initiation codons upstream of the main open reading frame 43. Such features may affect recognition and initiation from the correct start codon 40,41,44.

The earliest genetic evidence for specific cell cycle effects due to translational control was the isolation of budding yeast conditional mutants in what turned out to be translation initiation factors 5. One would expect that cessation of a continuous vital cellular function, such as initiation of translation, would simply arrest each cell at whichever point in the cycle that cell happened to be at the time. In an asynchronously proliferating cell population, this would manifest in a pattern of random arrests along the cell cycle 5. Yet *cell division cycle* (*cdc*) genetic screens yielded mutants carrying temperature-sensitive, hypomorphic alleles of translation initiation factors, which did not display a random arrest at their non-permissive temperature. Instead, cells carrying *cdc33* (encoding mRNA cap binding protein and translation initiation factor eIF4E 45,46) or *cdc63* (encoding the b subunit of translation initiation factor eIF3 47,48) mutations arrest uniformly in the G1 phase of the cell cycle, unable to initiate DNA replication and a new round of cell division 35,46,49,50. A conditional methionyl-tRNA synthetase (*mes1*) mutant also arrests in the G1 phase of the cell cycle 51. These classical genetic analyses suggested strongly that G1 transit is sensitive to translation initiation, more so than other phases of the cell cycle. This conclusion was strengthened when essential gene function was interrogated with a collection of titratable *TetO_7_* promoter alleles for essential genes 52. In addition to the eIF4E and eIF3b examples mentioned above, Yu *et al.* showed that inhibiting expression of eIF2a, eIF4A, eIF2b, eIF3i, or eIF1 resulted in G1 arrest in yeast (52; and Table 1). Hence, impairing translation initiation in a number of ways, invariably and specifically also impairs the capacity of cells to initiate a new round of cell division.

**Table 1:** Cell cycle phenotypes of loss-of-function mutants in essential genes encoding protein synthesis and ribosome biogenesis factors in *S. cerevisiae*.

**Systematic name**	**Standard name/ alias**	**Function**	**Cell cycle phenotype**	**Ref.**
**Translation Initiation Factors**				
*YER165W*	*PAB1*	Poly(A) binding protein	G1	136
*YJR007W*	*SUI2*	eIF2α	G1	52
*YKR059W*	*TIF1*	eIF4A	G1	52
*YLR291C*	*GCD7*	eIF2Bβ	G1	52
*YMR146C*	*TIF34*	eIF3i	G1	52
*YNL244C*	*SUI1/MOF2*	eIF1	G1	52
*YOL139C*	*CDC33/TIF45*	eIF4E	G1	45,46
*YOR361C*	*PRT1/CDC63*	eIF3b	G1	47,48
**Translation Elongation Factors**				
*YLR249W*	*YEF3*	eEF1Bγ	G2/M, other	52
**tRNA synthetases**				
*YGR264C*	*MES1*	MetRS	G1	51
*YLL018C*	*DPS1*	AspRS	G1	52
*YOR335C*	*ALA1/CDC64*	AlaRS	G1	50
*YPL160W*	*CDC60*	LeuRS	G1	50
**tRNA**				
*tQ(CUG)M*	*CDC65*	tRNA-Gln	G1	137
**Ribosome biogenesis and assembly**				
*YBL004W*	*UTP20*	18S rRNA biogenesis	other	52
*YBR142W*	*MAK5*	60S ribosome subunit biogenesis	G1	52
*YCL054W*	*SPB1*	AdoMet-dependent methyltransferase	G1	52
*YCR057C*	*PWP2/UTP1*	18S rRNA biogenesis	G1	108,138
*YDL031W*	*DBP10*	40S biogenesis and 35S pre-rRNA processing	G1	52
* YDL060W*	* TSR1*	20S pre-rRNA processing	G1	52
* YDL148C*	* NOP14/UTP2*	18S rRNA biogenesis	G1	138
* YDL153C*	* SAS10/UTP3*	18S rRNA biogenesis	G1, other	52,138
* YDL166C*	* FAP7*	20S pre-rRNA processing	G1	52
* YDR060W*	* MAK21*	60S ribosome subunit biogenesis	G1	52
* YDR091C*	* RLI1*	Ribosome biogenesis	G1	52
* YDR324C*	* UTP4*	18S rRNA biogenesis	G1	138
* YDR398W*	* UTP5*	18S rRNA biogenesis	G1	52,138
* YDR449C*	* UTP6*	18S rRNA biogenesis	G1	138
* YER006W*	* NUG1*	Export of 60S ribosomal subunits from the nucleus	G1	52
* YER082C*	* UTP7*	18S rRNA biogenesis	G1	52,138
* YER127W*	* LCP5*	18S rRNA maturation	G1	52
* YFL002C*	* SPB4*	60S ribosome biogenesis	G1	52
* YGR090W*	* UTP22*	18S rRNA biogenesis	G1	52
* YGR103W*	* NOP7*	60S ribosome subunit biogenesis	G1	52
* YGR128C*	* UTP8*	18S rRNA biogenesis	G1	52,138
* YGR245C*	* SDA1*	60S ribosome biogenesis and actin organization	G1	52
* YHR072W-A*	* NOP10*	18S rRNA maturation	G2/M	52
* YHR085W*	* IPI1*	35S pre-rRNA processing	G1	52
* YHR088W*	* RPF1*	Export of 60S ribosomal subunits from the nucleus	G1	52
* YHR089C*	* GAR1*	Modification and cleavage of the 18S pre-rRNA	other	52
* YHR143W-A*	* RPC10*	RNA polymerase subunit common to RNA polymerases I, II, and III	G2/M	52
* YHR196W*	* UTP9*	18S rRNA biogenesis	G1	52,138
* YJL033W*	* HCA4*	18S rRNA biogenesis	G1	52
* YJL069C*	* UTP18*	18S rRNA biogenesis	G1	52
* YJL109C*	* UTP10*	18S rRNA biogenesis	G1	138
* YJR002W*	* MPP10*	18S rRNA biogenesis	G1, other	52
* YKL009W*	* MRT4*	Ribosome assembly	G1	52
* YKL099C*	* UTP11*	18S rRNA biogenesis	G1	52,138
* YKL172W*	* EBP2*	25S rRNA maturation	G1	52
* YLL008W*	* DRS1*	DEAD-box protein, 60S ribosomal subunits	G1	52
* YLR002C*	* NOC3*	60S ribosome subunit biogenesis	G2/M	52
* YLR009W*	* RLP24*	60S ribosome subunit biogenesis	G1	52
* YLR129W*	* DIP2*	18S rRNA biogenesis	G1	52,138
* YLR167W*	* RPS31*	Ribosomal protein	G1, other	52
* YLR175W*	* CBF5*	Pseudouridine synthase	other	52
* YLR186W*	* EMG1*	Methyltransferase for rRNA	G1	52
* YLR222C*	* UTP13*	18S rRNA biogenesis	G1	138
* YLR276C*	* DPB9*	DEAD-box helicase, 27S rRNA processing	G1	52
* YML093W*	* UTP14*	18S rRNA biogenesis	G1	52,138
* YMR093W*	* UTP15*	18S rRNA biogenesis	G1	138
* YMR128W*	* ECM16*	DEAD-box helicase, 18S rRNA synthesis	G1	52
* YMR290C*	* HAS1*	Helicase, biogenesis of 40S and 60S ribosome subunits	G1	52
* YNL113W*	* RPC19*	RNA polymerase subunit common to RNA polymerases I and III	G1	52
* YNL124W*	* NAF1*	pre-rRNA processing	G1	52
* YNL163C*	* RIA1*	80S ribosome assembly	G1	52
* YNL207W*	* RIO2*	40S ribosome subunit biogenesis	G1	52
* YNR038w*	* DPB6*	DEAD-box helicase	G1	52
* YNR053C*	* NOG2*	60S ribosome subunit biogenesis	G1	52
* YOL010W*	* RCL1*	18S rRNA maturation	G2/M, other	52
* YOR078W*	* BUD21/UTP16*	18S rRNA biogenesis	G1	138
* YOR119C*	* RIO1*	40S ribosome subunit biogenesis	other	52
* YOR210W*	* RPB10*	RNA polymerase subunit common to RNA polymerases I, II, and III	G1	52
* YOR224C*	* RPB8*	RNA polymerase subunit common to RNA polymerases I, II, and III	G1	52
* YOR294W*	* RRS1*	Export of 60S ribosomal subunits from the nucleus	G1, other	52
* YOR340C*	* RPA43*	RNA polymerase I subunit	G1	52
* YOR341W*	* RPA190*	RNA polymerase I subunit	G1	52
* YPL012W*	* RRP12*	Export of ribosomal subunits from the nucleus	G1	52
* YPL043W*	* NOP4*	27S rRNA processing, 60S ribosome subunit biogenesis	G1, other	52
* YPL093W*	* NOG1*	60S ribosome subunit biogenesis	G1	52
* YPL126W*	* NAN1*	18S rRNA biogenesis	G1	52,138
* YPL211W*	* NIP7*	60S ribosome subunit biogenesis	G1, other	52
* YPL217C*	* BMS1*	40S synthesis and 35S pre-rRNA processing	G1	52
* YPL266W*	* DIM1*	18S rRNA dimethylase	G1	52
* YPR016C*	* TIF6/CDC95*	eIF6	G1	52
* YPR110C*	* RPC40*	RNA polymerase subunit common to RNA polymerases I and III	G1	52
* YPR144C*	* UTP19*	Maturation and nuclear export of 40S ribosomal subunits	G1	52

If initiation of translation is important for commitment to division, then signaling pathways that control initiation of division may do so, at least in part, by regulating translation initiation. Mitogenic pathways would be expected to activate translation initiation, while pathways that convey anti-proliferative signals may inhibit translation initiation. The cardinal example for the former case is the Target of Rapamycin (TOR) pathway. How the TOR pathway activates initiation of translation and overall protein synthesis has been reviewed elsewhere 53,54. Loss of TOR function was known to cause G1 arrest in mammals 55,56 and yeast 53,57. Connecting the G1 arrest with the effects of TOR on translation, however, was not obvious. In a landmark paper, it was shown that upon loss of TOR function in yeast, the cause of the G1 arrest was a direct consequence of a block in translation initiation 58. De-repressing translation of the G1 cyclin Cln3p was sufficient to abrogate the G1 arrest of TOR-inhibited yeast cells 58. TOR is not the only mitogenic pathway that activates translation initiation. The RAS/MAPK pathway in animals phosphorylates and increases the activity of eIF4B 40,59. Remarkably, phosphorylation of eIF4B on the same residue is a common output of both the TOR and MAPK pathways 59, underscoring the significance of activating translation initiation for commitment to cell division. Conversely, upon stress or starvation it is not prudent to either initiate cell division or make many proteins. It turns out that phosphorylation of eIF2α is a conserved response from yeast to mammals that inhibits overall translation initiation, and it is an output of anti-mitogenic signals 40,41,53,60.

## TRANSLATIONAL TARGETS (?) FOR COMMITMENT TO DIVISION

The above examples suggest that translation initiation goes hand-in-hand with G1 progression and initiation of cell division. By and large, however, they do not answer how this is brought about. What are the relevant proteins important for G1 transit, whose synthesis is sensitive to limitations in translation initiation, and how do these proteins impinge on the machinery of cell division? In the case of the G1 cyclin Cln3p was mentioned above, Hall and colleagues replaced the long 5’-UTR of the yeast *CLN3* mRNA with that of *UBI4*, which is efficiently translated when TOR function is low 58. Cells carrying this non-repressible *CLN3* did not arrest in G1 when TOR function was inhibited by rapamycin 58. Similarly, efficient translation of *CLN3* enabled G1 arrested *cdc33* cells, in which the activity of the eIF4E is impaired (see Table 1), to initiate cell division 61. The Whi3p RNA-binding protein, which sequesters *CLN3* mRNA in cytoplasmic foci, may inhibit translation of *CLN3* 62. There is also a uORF in the 5’-UTR of the *CLN3* mRNA 63. We had proposed that the uORF lowers the number of scanning ribosomes that reach the downstream main AUG, especially when the ribosome content of the cell is low in poor media 8,63. As predicted, inactivation of the uORF in *CLN3* allowed cells growing in poor medium, with glycerol as the source for carbon, to accelerate completion of START 63. Nitrogen limitation was also reported to repress translation of *CLN3* 64. In contrast, another study reported that 20 min after amino acid starvation, translation of *CLN3* was up-regulated 43. While this later discrepancy may simply reflect the different experimental set-ups, there is overall compelling evidence that synthesis of Cln3p, whose levels control the timing of START 65,66, is regulated at least in part at the translational level. It should be noted, however, that all the examples above rest on comparisons between different conditions: with or without inhibitors of TOR 58,63; mutant vs. wild type 61,62,63; different nutrients 43,63,64. It is important to stress that although *CLN3* is a translational target, there is no evidence yet that it is targeted in a periodic manner in the cell cycle, in G1 or any other cell cycle phase.

It has been reported that *CLN3* transcription oscillates early in the cell cycle 67,68, but Cln3p protein levels were not evaluated in these studies. Cln3p is very unstable 69, and difficult to detect by immunoblotting. Early studies reported that Cln3p levels do not oscillate in the cell cycle 70. Recently, however, more sensitive approaches from two independent studies showed that Cln3p protein is nearly absent in early G1 cells, but it gradually accumulates as cells approach START (Fig. 9 in 71, and Fig. 10 in 72), without a corresponding increase in the mRNA levels of *CLN3*. The data from Thorburn *et al.* 71 and Zapata *et al.* 72 strongly implicate post-transcriptional mechanisms that control abundance of Cln3p in the cell cycle, perhaps due to control of its synthesis, degradation, or both. Both of these studies relied on centrifugal elutriation to isolate highly synchronous early G1 daughter cells, which were then sampled as they progressed in the cell cycle 71,72. Until analogous studies are performed with alternative synchronization methods, it is formally possible that the results reflect idiosyncrasies of elutriation. Note also that the *CLN3* mRNA cannot possibly be the only physiological target of translational control for cell cycle progression. Cells lacking Cln3p are viable 65,66 and they also respond as expected to nutrient limitations, reducing their size 38.

In other systems, the best example of a translational target important for initiation of mitotic cell division is the cyclin-dependent kinase inhibitor p27^Kip1^ in human cells, whose translation appears to be periodic in the cell cycle, decreasing at the G1/S transition 73,74,75,76,77. Translational control of p27^Kip1^ is complex, involving both cap-dependent and independent mechanisms 75,77. However, because p27^Kip1^ synthesis *decreases* at the G1/S transition, this case of translational control cannot account for the postulated *activating* role of protein synthesis in triggering cell division. Other reported translational targets include the G1 cyclin D1 in mammals 78,79,80, and the G1/S cyclins E1 81 and E2 82 in mammals, and Cig2 in fission yeast 83 (reviewed in 84). Trans-acting factors influencing translation initiation of these targets include the helicase DDX3 for cyclin E1 81 and Ded1 for Cig2 83. It is not clear, however, if translation of these cyclins is periodic in cycling cells. Alternatively, their translational regulation may be an output of a continuous process that affects their overall levels. The levels of these cyclins may oscillate in the cell cycle for other reasons, such as mechanisms that control mRNA levels and protein degradation. Overall, there is a critical gap in our understanding of the role of translational control in mitotic cell cycle progression, especially in G1 progression and commitment to division. There have not been any studies that directly and systematically looked for mRNAs that are translated differentially in the G1 phase, in cycling, un-perturbed cells.

## START ON CYCLOHEXIMIDE

After the initiation step, the rate of translation depends on the concentration and activity of translating ribosomes that elongate the nascent polypeptides. If elongation of protein synthesis is inhibited, then what are the consequences on cell cycle progression? This question was first tackled pharmacologically, monitoring cell cycle progression in the presence of varying doses of cycloheximide 35,85,87,87. Cycloheximide inhibits the translocation step in eukaryotic 80S ribosomes, blocking translational elongation 88. Increasing doses of cycloheximide increase the population doubling time, mostly because cells spend more time in the G1 phase of the cell cycle 35,85. Cycloheximide also affects size homeostasis. In budding yeast, cycloheximide reduces the newborn cell size 35,85 and the rate at which cells increase in size 89. It also increases the critical size threshold for START 85,89. These changes account for the increase in the duration of the G1 phase upon treatment with cycloheximide. The effects of cycloheximide support the notion that a critical rate of protein synthesis is required for G1 transit and completion of START in budding yeast 86 and animal cells 90,91. The reports that interrogated cycloheximide’s effects on G1 progression have been influential. They have often been taken to imply a requirement for the continuous synthesis of unstable protein(s), whose rate of synthesis parallels overall protein synthesis rates and cell growth. Identifying those proteins and how they affect the cell division machinery would then hold the promise of explaining molecularly how cells couple their growth with their division 90,91. Various such candidate proteins have been proposed over the years. However, there is no report of a protein whose levels increase due to cell cycle-dependent translational control, as cycling cells approach START 7.

## RIBOSOME MAKES PROTEIN MAKES CELLS?

Ribosomes are the complex macromolecular machines that catalyze protein synthesis. Hence, changing the concentration of functional ribosomes in the cell is expected to change the overall rates of protein synthesis.

Bacterial cells grown under conditions that favor fast growth and proliferation (i.e., "rich" media) have more ribosomes than those propagated in "poor" media 92. These observations suggest that the rate of protein synthesis in bacteria is controlled mostly by ribosome numbers 93. Although growth rate does not seem to affect significantly the fraction of active ribosomes in the cell (≈80%) or their activity 94, recent observations suggest that even in rich media bacterial ribosomes do not function at maximal elongation rates 95. Hence, translation rates may be adjusted to proliferation rates by means other than ribosome content.

In budding yeast, although protein synthesis rates decrease with decreasing growth rates, there is no proportional decrease in the number of ribosomes per cell 96,97. For example, a 10-fold drop in growth rate is only accompanied by a 2-fold drop in the number of ribosomes per cell (see Table 4 in 96). On the other hand, synthesis of ribosomal components is inhibited as cells begin to exhaust the available nutrients and prepare to enter stationary phase 98, or upon amino acid starvation 99. Apparently, both the activity and the number of ribosomes may be affected in yeast as a function of growth rate.

In cycling, unperturbed yeast cells, synthesis of ribosomal components is not cell cycle dependent 10,22,23. Interfering with ribosome biogenesis, however, affects cell cycle progression dramatically. In yeast, 59 of a total of 78 ribosomal proteins of cytoplasmic ribosomes are encoded by pairs of very similar or identical paralogous genes 100. Mutants carrying single deletions of those ribosomal protein genes are usually viable 101. Many of these deletion strains have a small overall cell size 102,103. A small overall cell size is also characteristic of cells lacking Sfp1p, a transcriptional activator of ribosome biosynthesis and ribosomal protein genes 89. Cells lacking Sfp1p are born very small 104, and their growth rate is about half of that of wild type cells 71,104. As a result, the duration of the G1 phase of the cell cycle is greatly increased in* sfp1*Δ cells 71,89,104. Still, the smaller critical size threshold of *sfp1*Δ cells and the smaller overall size of ribosomal protein mutants led Tyers and colleagues to propose that ribosome biogenesis *inhibits* START in wild type cells 6,89. In this model, ribosome biogenesis sets the critical size threshold for START, while translation rates of functional ribosomes enable the cells to pass that threshold 6,89. This model appears paradoxical and counterintuitive, especially since in animals ribosome biosynthesis is thought to promote cell proliferation and cancer 105. But another finding, that ribosomal proteins may function as haploinsufficient tumor suppressors in animals, appeared to offer support for a negative role of ribosome biogenesis in cell division 106. However, it was subsequently reported that such effects may be due to cell non-autonomous routes 107. Furthermore, the following observations argue against the notion that ribosome biogenesis has a general inhibitory role for the initiation of division: First, inhibition of ribosome maturation delays START 108. Second, despite their small cell size, most ribosomal protein mutants have a longer G1 phase 104,109,110. Third, loss-of-function of the vast majority of essential ribosome biogenesis factors leads to G1 arrest (see Table 1 and 52). The most straightforward interpretation of the evidence outlined above is that ribosome biogenesis *promotes* initiation of cell division in budding yeast.

Even when there is a G1 delay due to loss of a ribosomal protein, G1 variables such as birth and critical size of cells lacking individual ribosomal proteins are not uniform, differing qualitatively and quantitatively 104,109,111. Furthermore, although a delay in G1 is the most common phenotype upon loss of a ribosomal protein, this phenotype is not universal. In several cases, there is no cell cycle phenotype, or a G2/M block is observed instead 104,110. The basis of all those differences in ribosomal protein mutant phenotypes related to the cell cycle is not clear. Do they reflect specialized translational roles of some ribosomes? Are overall translation rates affected? Is the concentration of ribosomes, their composition, their activity, localization in the cell (or any combination of the above) that is affected? Examples of specialized ribosomal functions abound 112,113,114,115, leading some to speculate on the existence of a “ribosome code” 116. There are even cases of extraribosomal roles for ribosomal proteins 117. Regardless of the answers to the above questions, it is imperative that the relevant mRNA substrates affected in each ribosomal protein mutant must be identified, to understand how cell cycle progression might be impacted. However, as is the case for wild type cells (see *INITIATE TO START?* above), there are no systematic surveys of mRNAs that are translated differentially in the cell cycle in ribosomal protein mutants.

## THE OTHER RIBOSOME SUBSTRATES: tRNAS

For the ribosome to elongate at its maximal rate, all its substrates must be present at saturating concentrations. This includes the aminoacyl-tRNAs and the various elongation factors 95. Inhibition of elongation factor eEF2 may be one way that protein synthesis is inhibited in animal cells in mitosis 118. However, unlike the numerous examples of perturbations in translation initiation factors that lead to G1 arrest in yeast (Table 1), there is very little analogous evidence for translation elongation factors affecting cell cycle progression. The only reported example is from a large survey of essential genes, reporting that blocking expression of eEF1Bγ in yeast leads to cell cycle arrest in mitosis (52, see Table 1).

Translation elongation rates may be affected by several parameters 119, including the supply and demand for each tRNA. The genetic code is essentially universal. One and the same codon does not code for different amino acids in different organisms. The code, however, is also degenerate. Most amino acids can be specified by more than one codon. A decades-old speculation has been that codons for an amino acid that are used more frequently in an organism would be translated more rapidly than codons for the same amino acid that are rarely used 120. Differences in codon usage were difficult to test until the advent of ribosome profiling. Sequencing ribosome-protected mRNA fragments allows estimates of ribosome residency on individual codons 43. Several analyses that included the original ribosome profiling datasets in yeast 43 have examined various parameters, including codon usage, and reached contradictory conclusions 42,119,121,122,123,124,125,126. Nonetheless, common codons may be translated faster than rare ones in yeast 127. Modulating which codon is used and the effective concentration of its corresponding tRNA may be a way to influence translation elongation rates both within an mRNA, and across the transcriptome 128. Surveying codon usage across each codon and tRNA availability revealed that in highly expressed mRNAs rare codons are more prevalent in the first 30-50 codons, slowing down and crowding ribosomes along that stretch 129. It was proposed that this slow “ramp” is a mechanism that promotes faster translation downstream, by alleviating ribosome “traffic jams” along the mRNA 129. Furthermore, it turns out that once a particular codon has been used, it will also be used more frequently whenever the same amino acid is encoded downstream in that mRNA 130. Based on these observations, it was theorized that tRNAs do not diffuse away from the ribosome once they are expelled from it. Instead, the same tRNA for a codon specifying the repeating amino acid is re-charged and channeled for re-use, enabling translation to proceed faster than tRNA diffusion 130.

The examples outlined above illustrate how alterations in the supply and demand for each tRNA could impact translation rates. The pertinent question for this review, however, is whether such mechanisms could lead to translational control during the cell cycle. Remarkably, a recent study proposed exactly that kind of regulation, based on codon usage 131. Optimal codon usage is more prevalent in mRNAs expressed in the G1 phase of the cell cycle, implying that the corresponding mRNAs are translated more efficiently 131. Interestingly, it has been known for decades that loss-of-function mutations in several tRNA synthetases, and even in a tRNA gene, lead to G1 arrest in yeast (see Table 1). Optimizing translation elongation rates in G1 through codon usage is an exciting possibility. But a correlation is not causation, and the above predictions must await experimental validation, measuring the translational efficiencies of the putative mRNA targets in cycling cells. On a cautionary note, a recent study in mouse embryonic stem cells reported that translation elongation rates were not only independent of codon usage, but also very similar across different mRNAs 132. Likewise, another study in yeast reported that the rate of translation elongation and translational efficiency were not affected by tRNA abundance, and codon translation rates were not correlated with codon bias 133. Hence, these issues remain controversial and there might be some time before the dust settles.

## DIVIDE TO TRANSLATE? 

Implicit in the discussion of all the examples mentioned above is the notion that cell growth and protein synthesis drive cell cycle progression, not the other way around. This is generally the case, established in classic and particularly lucid experiments by Hartwell and colleagues 4,5,35. However, there are also some changes in the pattern of growth once cell cycle progression is blocked 134,135. Does that mean that progressing in the cell cycle might also affect the translational control of specific mRNAs? The answer is a resounding yes. Ruggero and colleagues examined by ribosome profiling human cells arrested by thymidine block (G1 and S phases) and nocodazole treatment (G2 phase) in the cell cycle, reporting extensive translational control of numerous mRNAs 17. Interestingly, this work revealed functional clusters of co-regulated mRNAs. Translational control may be used to coordinate expression of specific cellular machines and processes 17. These experiments are very important, answering how cell division controls translation. However, they do not answer how translation controls cell division.

## OUTLOOK

The role of protein synthesis and translational control has a long history in the cell cycle field. The pioneering experiments of decades ago were incisive, but also largely descriptive. Since then, progress has been incremental and focused on a limited number of putative mRNA translational targets. Identifying all the mRNAs that are under periodic translational control in cycling cells is an obvious and necessary goal. Given the transformative methodologies now available and the current pace of progress, it is only a matter of time before we know how protein synthesis drives cell cycle progression.
